# Genome-wide identification and stress response analysis of BcaCPK gene family in amphidiploid *Brassica carinata*

**DOI:** 10.1186/s12870-024-05004-9

**Published:** 2024-04-17

**Authors:** Dan Zuo, Shaolin Lei, Fang Qian, Lei Gu, Hongcheng Wang, Xuye Du, Tuo Zeng, Bin Zhu

**Affiliations:** 1 School of Life Sciences, Guizhou Normal University, Guiyang, 550025 China; 2 Guizhou Institute of Oil Crops, Guizhou Academy of Agricultural Sciences, Guiyang, 550009 China

**Keywords:** *Brassica carinata*, Genome-wide, Calcium-dependent protein kinases (CPKs), Bioinformatics analysis, Stresses

## Abstract

**Background:**

Calcium-dependent protein kinases (CPKs) are crucial for recognizing and transmitting Ca^2+^ signals in plant cells, playing a vital role in growth, development, and stress response. This study aimed to identify and detect the potential roles of the *CPK* gene family in the amphidiploid *Brassica carinata* (BBCC, 2n = 34) using bioinformatics methods.

**Results:**

Based on the published genomic information of *B. carinata*, a total of 123 *CPK* genes were identified, comprising 70 *CPK* genes on the B subgenome and 53 on the C subgenome. To further investigate the homologous evolutionary relationship between *B. carinata* and other plants, the phylogenetic tree was constructed using CPKs in *B. carinata* and *Arabidopsis thaliana*. The phylogenetic analysis classified 123 family members into four subfamilies, where gene members within the same subfamily exhibited similar conserved motifs. Each BcaCPK member possesses a core protein kinase domain and four EF-hand domains. Most of the *BcaCPK* genes contain 5 to 8 introns, and these 123 *BcaCPK* genes are unevenly distributed across 17 chromosomes. Among these *BcaCPK* genes, 120 replicated gene pairs were found, whereas only 8 genes were tandem duplication, suggesting that dispersed duplication mainly drove the family amplification. The results of the Ka/Ks analysis indicated that the *CPK* gene family of *B. carinata* was primarily underwent purification selection in evolutionary selection. The promoter region of most *BcaCPK* genes contained various stress-related *cis*-acting elements. qRT-PCR analysis of 12 selected *CPK* genes conducted under cadmium and salt stress at various points revealed distinct expression patterns among different family members in response to different stresses. Specifically, the expression levels of *BcaCPK2.B01a*, *BcaCPK16.B02b*, and *BcaCPK26.B02* were down-regulated under both stresses, whereas the expression levels of other members were significantly up-regulated under at least one stress.

**Conclusion:**

This study systematically identified the *BcaCPK* gene family in *B. carinata*, which contributes to a better understanding the *CPK* genes in this species. The findings also serve as a reference for analyzing stress responses, particularly in relation to cadmium and salt stress in *B. carinata*.

**Supplementary Information:**

The online version contains supplementary material available at 10.1186/s12870-024-05004-9.

## Background

As a crucial second messenger in plant cells, Ca^2+^ plays a significant role in various essential processes such as plant growth and development, environmental response, and stress response [1]. The concentration of Ca^2+^ differs in different cellular compartments, and it remains relatively stable in a static state. However, the osmotic pressure of the cell membrane increases when the cell senses a stress signal, such as adversity stress, leading to the activation of calcium channels through phosphorylation reactions. Consequently, the concentration and distribution of free Ca^2+^ within the intracellular space and concentration change, resulting in the generation of calcium signals [2]. Five calcium sensing proteins have been identified in plants: calmodulin (CaM), calmodulin-like protein (CML), calcineurin B-like protein (CBL), calcium, calmodulin-dependent protein kinase (CCaMK), and calcium-dependent protein kinase (CPK) [3]. Among these, only calcium-dependent protein kinases (CPKs) have the ability to directly convert Ca^2+^ signals into phosphorylation cascades, thus serving as both Ca^2+^ sensors and responders [4].

CPK protein consists of four distinct domains: the N-terminal variable region, the Ser/Thr protein kinase catalytic region, the activity-controlled self-inhibiting junction region, and the CaM regulatory region containing the EF-hand motif responsible for Ca^2+^ binding [5]. These unique structural components enable CPK to function as a sensor and effector in calcium signaling [4]. CPKs play a crucial role in regulating stress signals, hormone responses, and metabolic pathways, and are widely distributed in various organs. While some CPKs are found universally, others are specific to certain conditions and tissues [6]. Previous studies have reported the essential roles of *AtCDPK17* and *AtCDPK34* in *Arabidopsis thaliana* in pollen suitability and the enhancement of pollen tube tip growth through the transmission of Ca^2+^ signals [7]; Overexpression of *OsCDPK2* in rice (*Oryza sativa*) has been found to disrupt normal seed development, suggesting that the CPK is crucial to seed formation [8]. Additionally, CPK proteins have been shown to play important roles in responding to abiotic stress. For instance, *OsCDPK4* has been identified as an important player in drought and salt stress in rice [9]. Both silencing and overexpression experiments of *OsCDPK9* have demonstrated its positive regulatory function in drought stress [10]. In both rice and *A. thaliana*, *OsCPK7*, *OsCPK13*, and *AtCPK1* are involved in the response to low temperature stress response [11, 12], *Populus euphratica PeCPK10* and *Vitis amurensis VaCPK20* have been found to positively regulate the response to low temperature stress response [13, 14]. However, *ZmCPK1* in maize (*Zea mays*) has been shown to have a negative regulatory role in low temperature signal transduction [15].

The amphidiploid *B. carinata* (BBCC, 2n = 34), commonly known as Ethiopian rape, which has originated from natural chromosome doubling after hybridization between diploid *B. oleracea* (CC, 2n = 18) and *B. nigra* (BB, 2n = 16) [16], has cultivated as a biofuel crop for its desirable seed fatty acid profile in Africa for a long history [17]. In addition, *B. carinata* has been found to harbour various resistant traits to various biotic and abiotic stresses [18, 19] because this species has not fully undergone artificial domestication. It is reported that *B. carinata* is one of the most drought and heat tolerant species within the Brassicaceae family. Recently, a high quality genome of *B. carinata* has been released [20], facilitating to analyze dynamic evolution and functions of some important gene family.

*B. carinata* and other five commonly known species (*B. rapa*, *B. oleracea*, *B. nigra*, *B. juncea*, and *B. napus*) in *Brassica* constituted.the famous triangle of U, which comprehensively describes the evolution and relationships among the six *Brassica* species [21]. Although the *CPK* gene family of other five *Brassica* species has been deciphered, the distribution and function of this gene family are still unclear in *B. carinata*. To understand the comprehensive information and potential function of *CPK* genes in *B. carinata* (*BcaCPK*), this study fully analyzed the *CPK* gene family by conducting a bioinformatics search of the whole genome database. Additionally, the evolutionary relationship of the *CPK* gene was analyzed through the construction of a phylogenetic tree, gene structure analysis, chromosome localization, and replication event analysis. Both transcriptome data and qRT-PCR analysis showed that some *BcaCPK* genes were likely involved in response to abiotic stresses. The findings of this study can serve as a valuable reference for further understanding the *BcaCPK* and its biological function in stress responses.

## Results

### Identification and bioinformatics analysis of BcaCPK genes

After merging and excluding the redundant sequences, a total of 123 *BcaCPK* genes and named based on its chromosome location, comprising of 70 *CPK* genes on B subgenome and 53 on C subgenome, were identified using protein sequence alignment and conserved domain screening (Table S1). Based on the bioinformatics analysis, these BcaCPKs are hydrophilic proteins with gene lengths ranging from 2,158 to 69,414 bp. They encode protein lengths ranging from 405 to 846 aa and protein molecular weights ranging from 45.49 to 95.21 kDa. The protein length and molecular weight varied significantly among the members. Approximately 10.5% of the members were alkaline proteins, while the remaining members had isoelectric points less than 7, making them acidic proteins. Furthermore, 47.2% of the BcaCPK members were classified as unstable proteins, whereas the other members were considered stable proteins. Subcellular localization prediction results showed that BcaCPK was localized in the nucleus. Protein structure analysis revealed that all BcaCPKs contained four EF-hand structures. Further analysis of palmitoylation and myristoylation sites indicated that, except for BcaCPK2.B01a, BcaCPK26.B02, BcaCPK11.B03, BcaCPK26.B03, BcaCPK26.B05, BcaCPK33.B06, BcaCPK4.B08, BcaCPK11.C03, and BcaCPK11.C05, the remaining 114 BcaCPKs contained palmitoylation sites. Additionally, 70 BcaCPKs were found to have myristoylation sites (Table S2).

### Phylogenetic analysis of BcaCPKs and AtCPKs

**Fig. 1:**
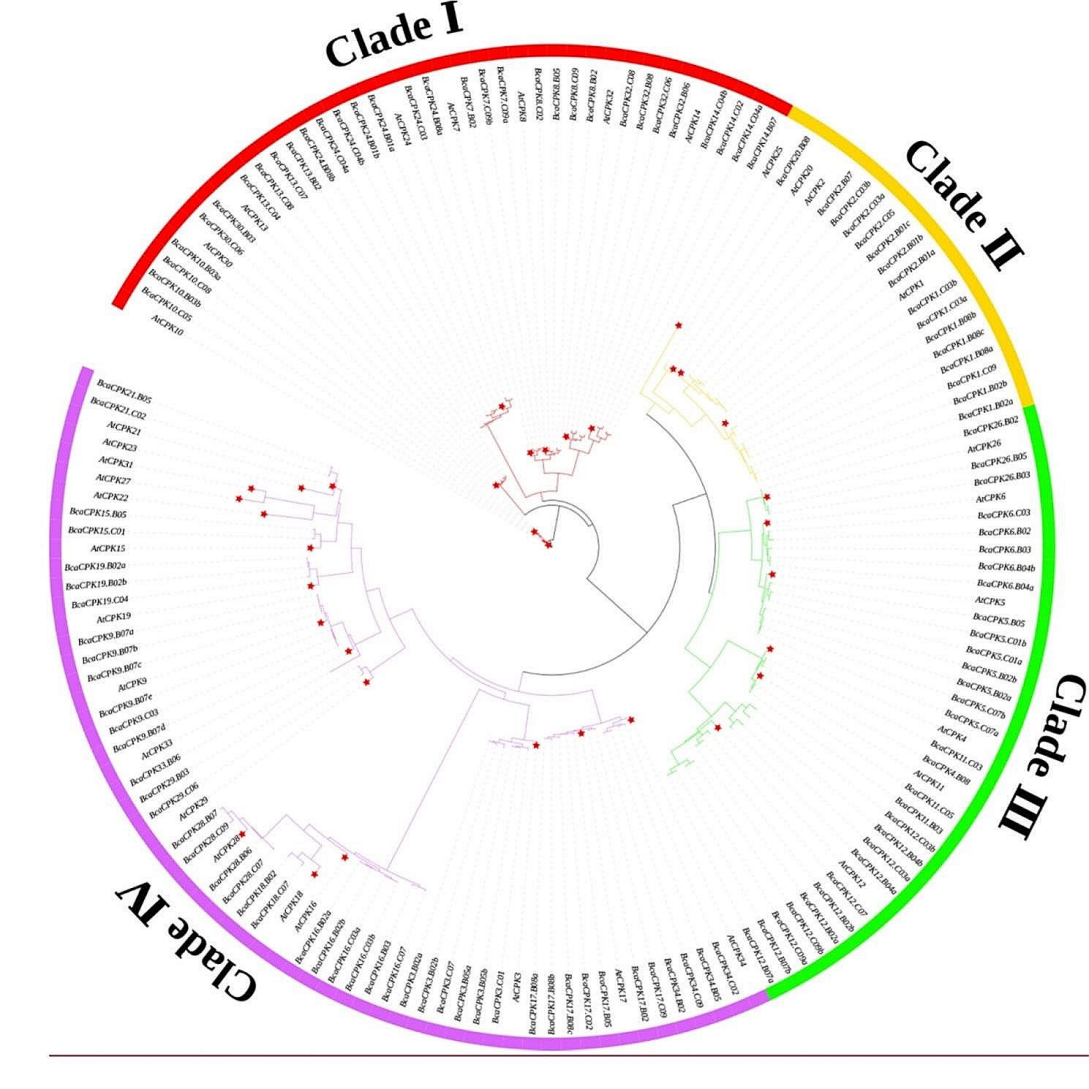
Phylogenetic tree of (**A**) thaliana and (**B**) carinata CPKs. The ML tree was constructed using MEGA 11 software based on the CPK protein sequences

To better understand the evolutionary relationship of these BcaCPKs, we constructed the phylogenetic tree of CPK proteins in *B. carinata* and *(A) thaliana* was constructed using MEGA software. The results revealed that the CPK proteins in both species could be categorized into four subfamilies, namely Clade I, Clade II, Clade III, and Clade IV (Fig. 1). The number of gene members varied significantly among these subfamilies. Clade II had the lowest number of members, with only 16 BcaACP family members, while Clade IV had the highest member, with 45 BcaACP family members. In *Arabidopsis*, the distribution of AtCPKs members across the four subfamilies was 8, 4, 6, and 16, respectively, with Clade II having the fewest members and Clade IV having the most. Similarly, in *(B) carinata*, the number of gene members in Clade I-IV was 32, 16, 30, and 45, respectively, showing a distribution pattern similar to that of *A. thaliana*.

### Structure analysis of BcaCPKs

**Fig. 2:**
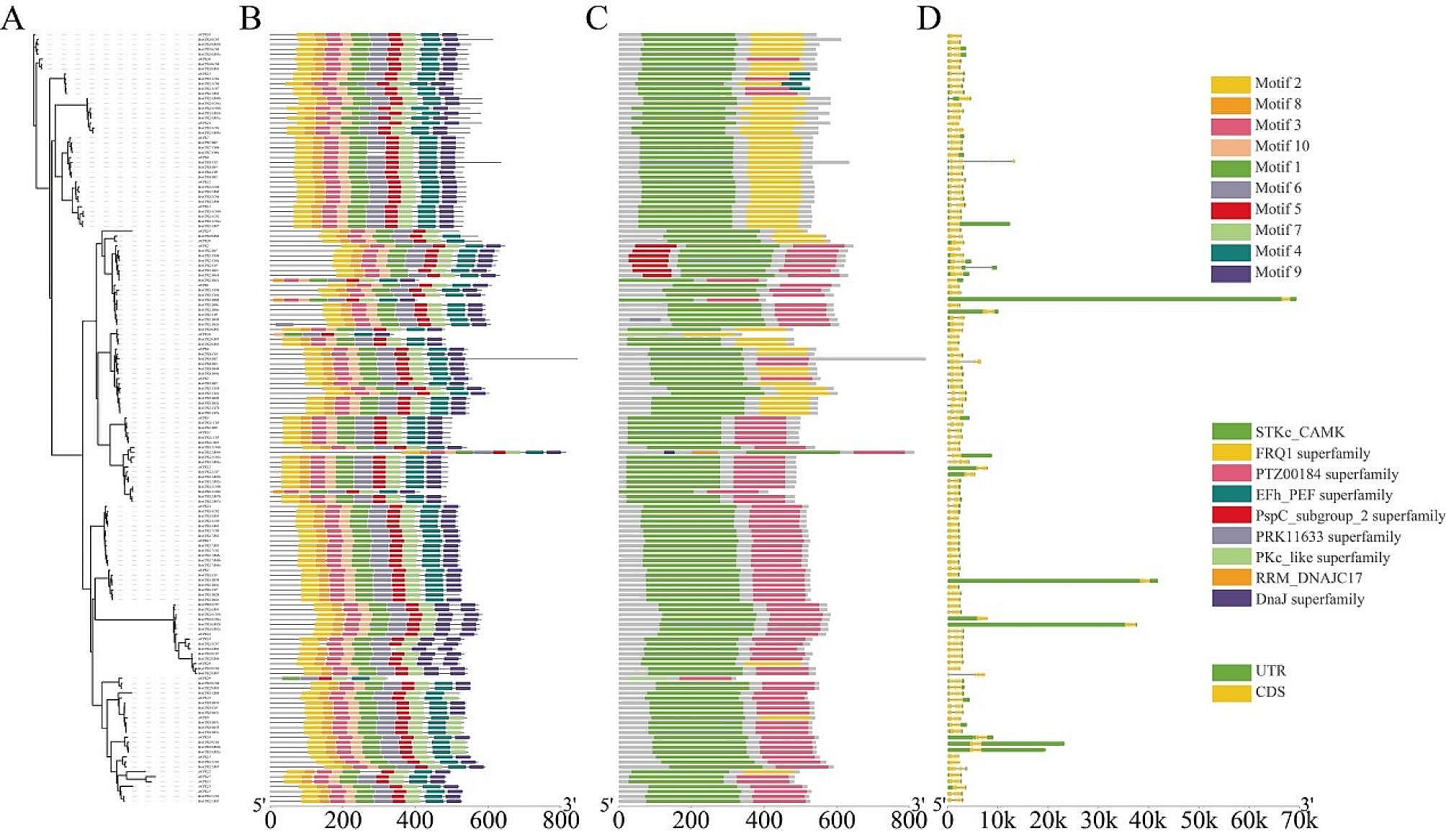
Detailed structure of CPK family proteins in (**A**) thaliana and (**B**) carinata. (**A**) Phylogenetic tree, (**B**) conserved motif, (**C**) conserved domain, (**D**) gene structure

In total, 10 conserved motifs were obtained in BcaCPKs using MEME online software. These motifs were named as Motif 1 to Motif 10 in accordance to the degree of conservation from high to low (Fig. 2A and B). The analysis found that the BcaCPK family protein sequence was highly conserved, and the motif covered most of the protein sequences. All members of the Clade I subfamily contain 10 Motifs, which proves that the protein of this subfamily is more conservative. The conserved domains of the family members were analyzed by CDD, and the results showed that each family member contained a core protein kinase domain (Pkinase) and four EF-hand domains (Fig. 2C). Through the analysis of gene structure, it was found that the number of introns of *BcaCPK* gene was quite different, and the number of introns ranged from 5 to 13. The most was *BcaCPK12.B04a*, with 13, and the least was 5. In addition, the number of introns in Clade I and Clade II subfamilies is mainly 6, which proves that Clade I and Clade II subfamilies are relatively conservative members. Clade III has the largest difference in gene structure, which contains the most and least introns of *BcaCPKs* (Fig. 2D).

### Chromosomal localization analysis of BcaCPKs

**Fig. 3:**
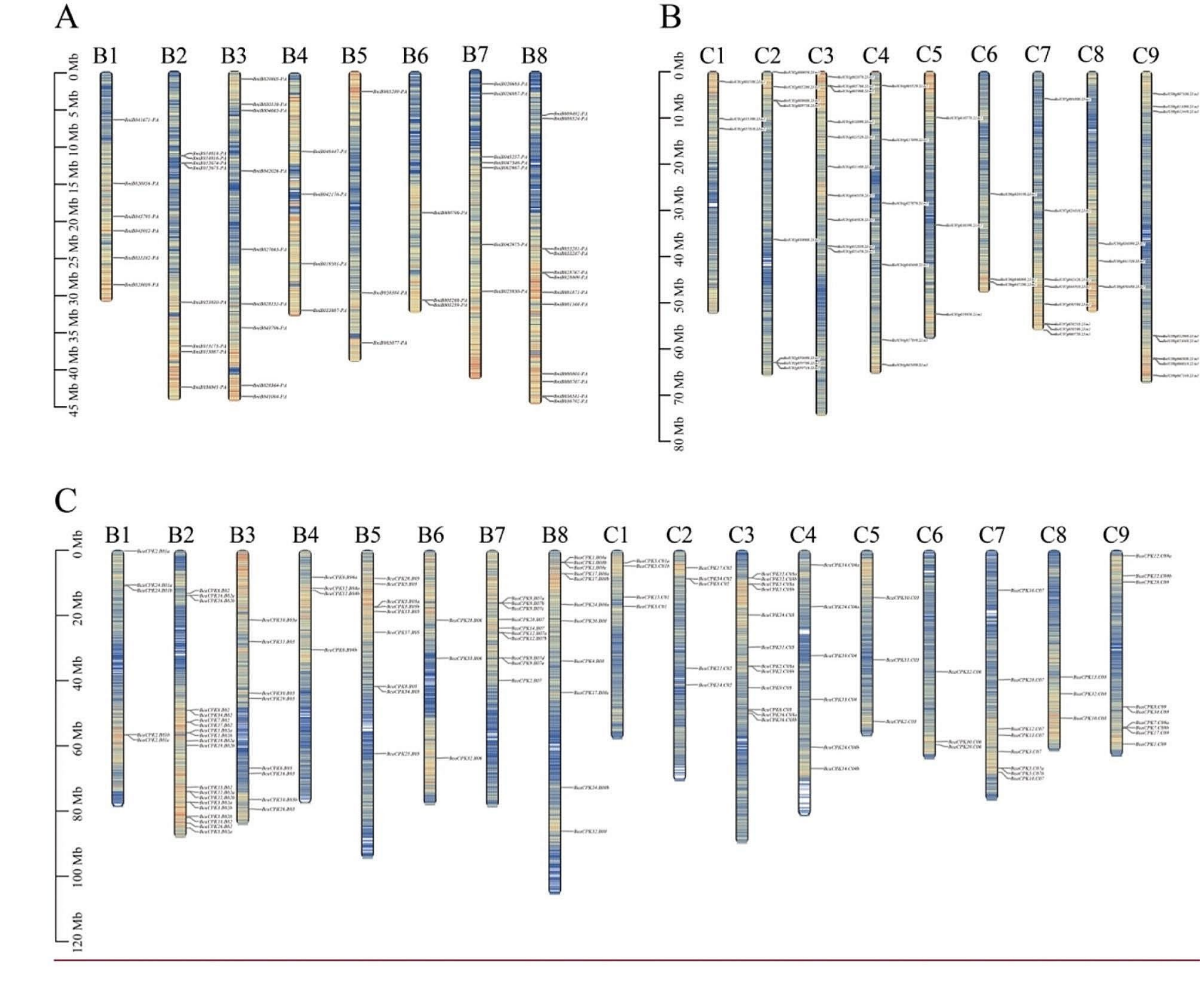
The distribution of *CPK* genes on chromosome in diploid ancestral species and allotetraploid *B. carinata*

To compare the divergence of *CPK* genes between *B. carinata* and it diploid parents, the location and distribution of these *CPK* genes on chromosomes were visualized based on the assembled genome information. The results exhibited that a total of 227 *CPK* genes were identified in three *Brassica* species, including 52 genes in *B. nigra*, 52 in *B. oleracea*, and 123 in *B. carinata* (70 in B subgenome and 53 in C subgenome). These *CPK* genes were not evenly distributed on the chromosomes (Fig. 3). For example, 20 *BcaCPK* genes were identified on B2 chromosome, but only 3 *BcaCPK* genes were found on B6, C5, C6, and C8 chromosomes, respectively. When compared to the gene distribution in the two diploid species, we found that most of the *CPK* genes in the allotetraploid *B. carinata* exhibited conserved chromosomal location after polyploidization. Furthermore, we found that some *CPK* genes in *B. carinata* have expanded in the B subgenome, especially the genes on the B2 chromosome (There are 8 *BniCPK* genes on the B2 chromosome of *B. nigra* and 20 *BcaCPK* genes on the B2 chromosome of *B. carinata*), which is likely attributed to chromosomal segment duplication along with the diploidization of allotetraploid *B. carinata*.

### Colinearity analysis of BcaCPKs

**Fig. 4:**
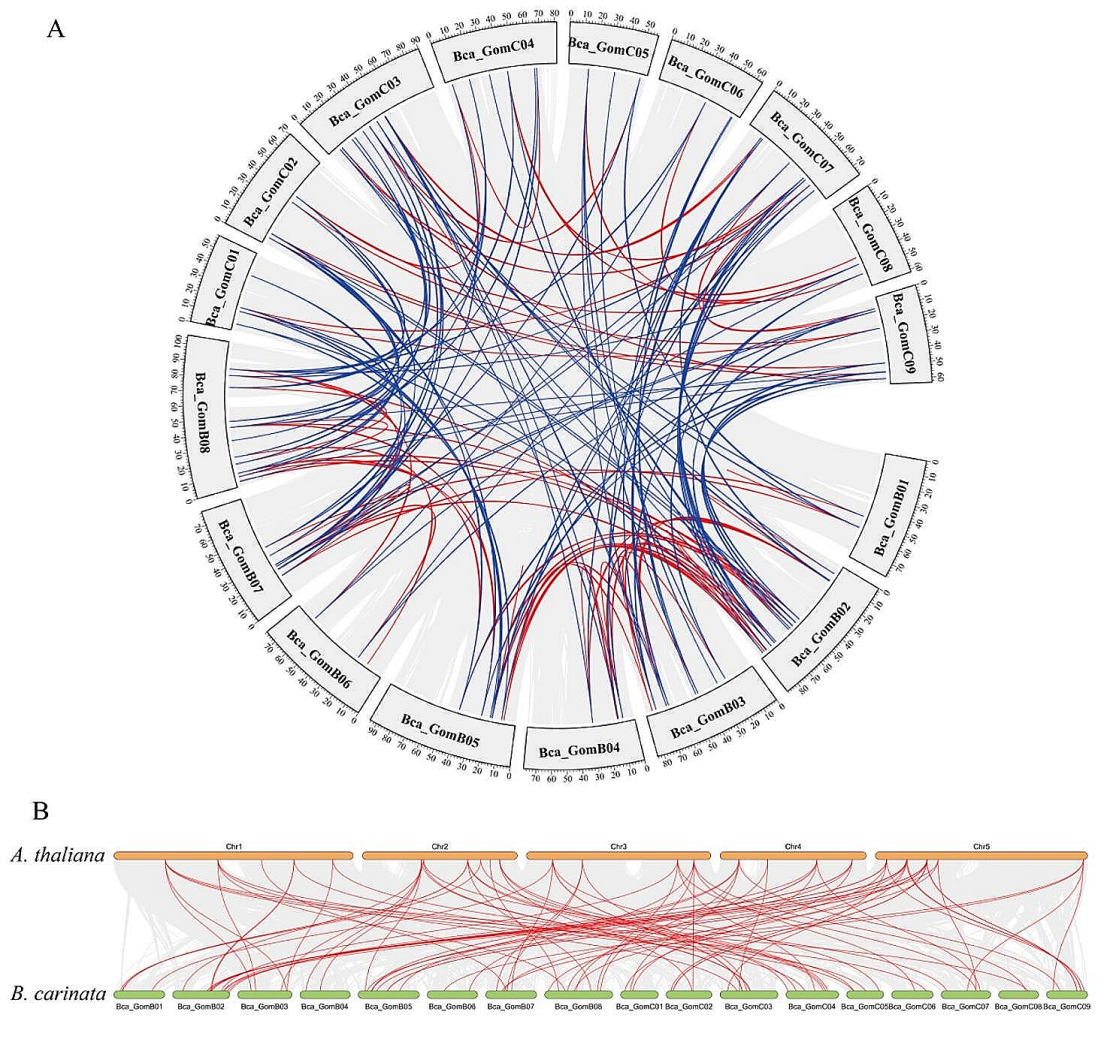
Collinearity analysis of *CPK* genes in *B. carinata*. (**A**) The red line represents the collinearity gene within the subgenome, and the blue represents the collinearity gene between the subgenomes, (**B**) the red line represents the collinearity gene between the *A. thaliana* and *B.carinata* genomes

To further check the driving force of the expansion of *CPK* genes in *B. carinata*, we detected the intraspecific and interspecific collinearity gene pairs among these *CPK* genes in *B. carinata* and *A. thaliana* using JCVI v1.3.5 [22]. The red line in Fig. 4A represents the collinearity gene within the subgenome, and the blue represents the collinearity gene between the subgenomes, the red line in Fig. 4B represents the collinearity gene between the *(A) thaliana* and *B.carinata* genomes, and the genes connected by the same color line represent the same type of *CPK* gene. Therefore, we can see that many chromosomes in all genomes/subgenomes are connected by the same color lines, indicating that these genomes/subgenomes are evolutionarily related. Subsequently, we employed the DupGen _ finder [23] to screen the gene duplication in *BcaCPKs.* The results exhibited that a total of 120 pairs of duplicated gene pairs were found in these *BcaCPKs*, including an overrepresented dispersed duplication (DSD), which were occurred between different chromosomes and only two pairs of tandem duplication (TD) sequences (Table S3). The results suggest that DSD events had a great driving force for the *CPK* expansion in *(B) carinata*.

### Ka/Ks analysis of BcaCPKs

**Fig. 5:**
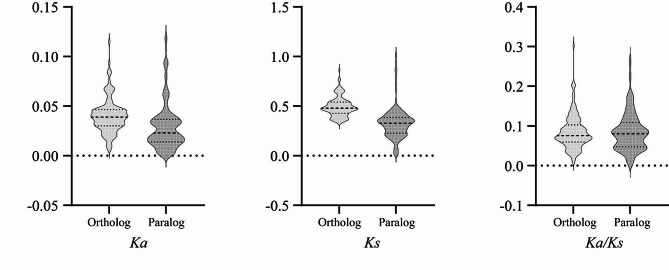
Ka/Ks values of *CPK* genes in *B. carinata*

The ratio of Ka/Ks is used to determine whether there is selective pressure on the gene encoding the protein. The results showed that only one genes of *CPK* gene family in *B. carinata* had Ka/Ks greater than 1, indicating that they were strongly positively selected. A gene pair Ka/Ks is greater than 0.5 and less than 1, indicating that the gene pair is weakly positively selected; the Ka/Ks of the remaining 98% gene pairs were less than 0.5, indicating that these genes were subjected to purification selection (Fig. 5, Table S4). It can be seen that the *CPK* gene family of *B. carinata* is mainly affected by purification selection in evolutionary selection.

### Prediction of cis-acting elements of BcaCPKs

**Fig. 6:**
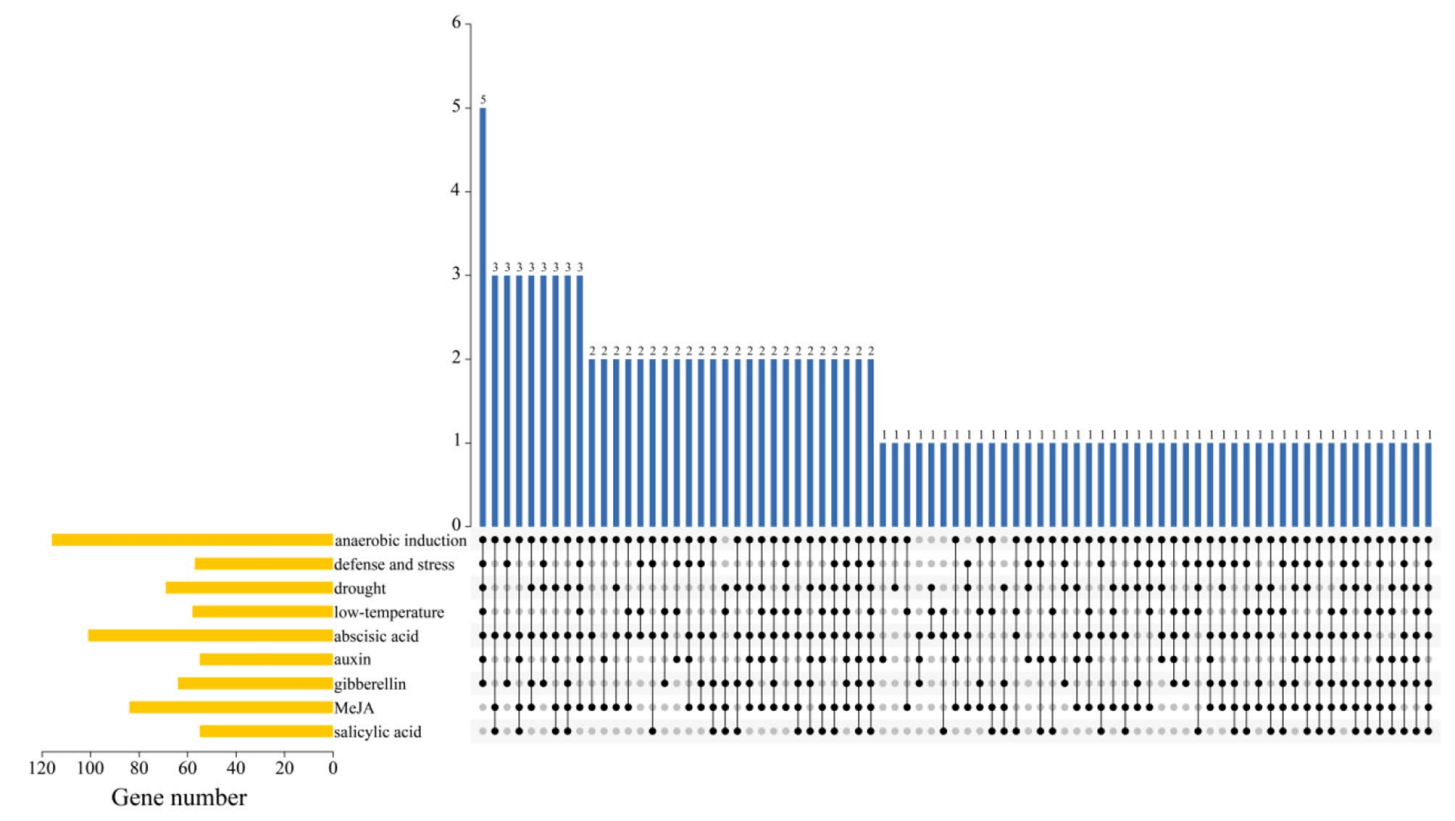
The prediction results of the *cis*-acting elements of the *CPK* gene family in *B. carinata*

To further understand the possible functions of *BcaCPKs*, we used PlantCARE online software (http://bioinformatics.psb.ugent.be/webtools/plantcare/html/) to determine the *cis*-acting elements in the 2 kb sequence upstream of the transcription start site of these genes. We found that these *cis*-elements in *BcaCPKs* were overrepresented by some elements (Fig. 6) responding to various abiotic stresses, such as LTR, MBS, GC-motif, TC-rich repeats, O2-site, and AuxRR-core, indicating that *BcaCPKs* likely play an important role in responding to abiotic stresses.

### Expression patterns of BcaCPKs under cadmium stress of RNA-seq data

**Fig. 7:**
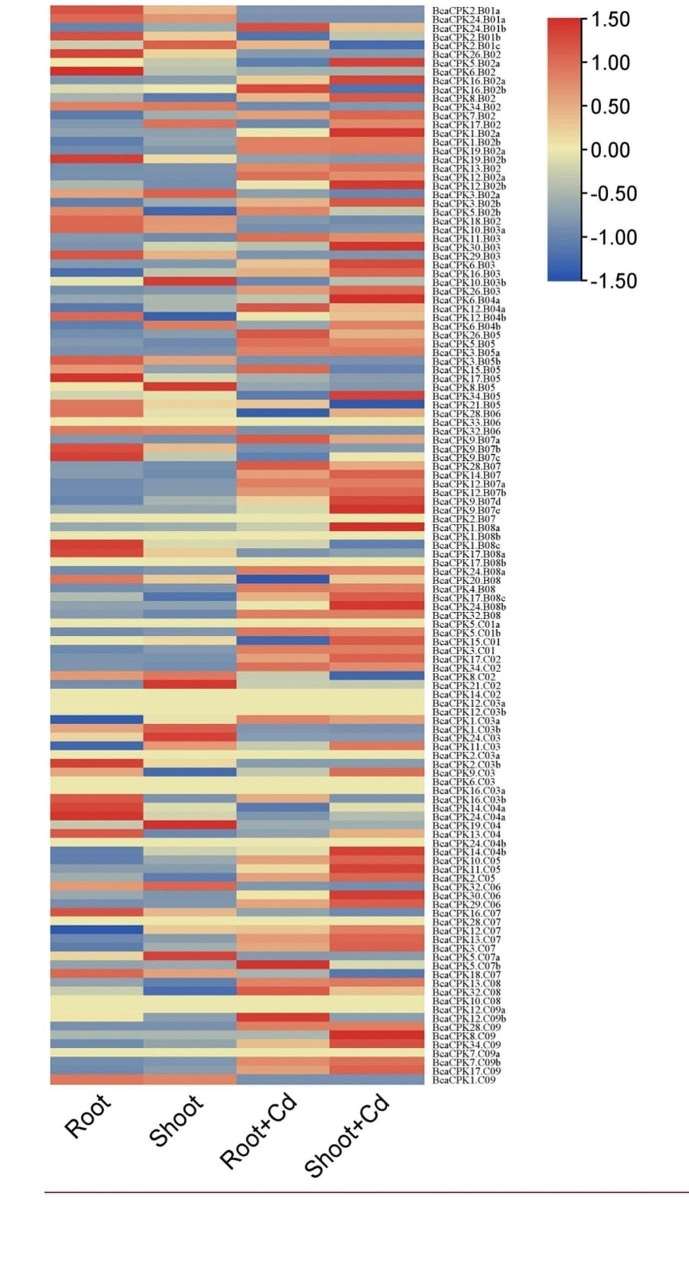
Expression analysis of *CPKs* in *B. carinata* under different tissues and cadmium stress. After standardizing the expression data with log_2_(FPKM), TBtools is used to visualize the expression data

We based on the previously determined RNA-seq data of *B. carinata* under cadmium stress and constructed a heatmap of *BcaCPKs* in root and shoot (Fig. 7). The results showed that these *BcaCPKs* exhibited obviously different expression patterns between root and shoot. We noticed that the expression of the majority of *BcaCPKs*, particularly these *BcaCPKs* in shoot, showed significantly upregulated after exposing to cadmium, suggesting that some *BcaCPKs* (such as *BcaCPK5.B02a*, *BcaCPK19.B02a*, *BcaCPK24.B01b*, and *BcaCPK28.B07*, etc.) involved in the responding to cadmium stress in *B. carinata*.

### Expression profiles of BcaCPKs under cadmium stress using qRT-PCR

**Fig. 8:**
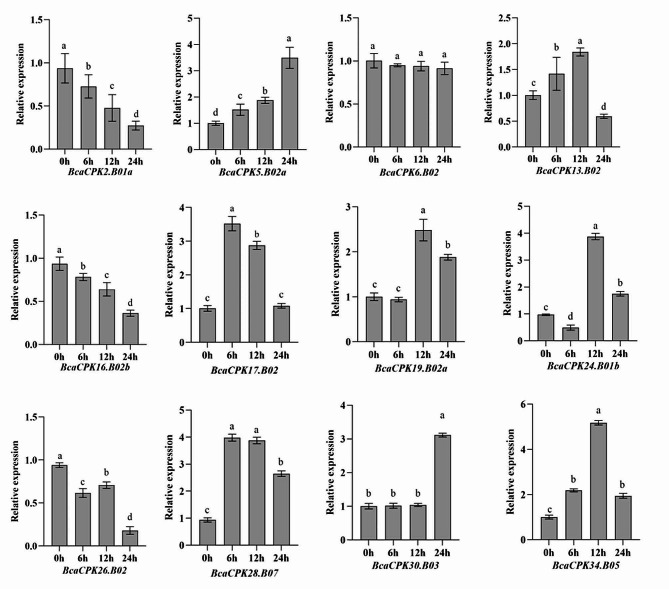
The relative expression of selected *BcaCPK*s under the treatment of 150mM/L CdCl_2_. Different letters indicate a statistically significant difference at *P* < 0.05

To further confirm *BcaCPKs* response to cadmium stress, the expression profile of 12 *BcaCPKs* exhibiting differential expression under cadmium stress based on the RNA-seq data were detected using qRT-PCR. These genes expression was determined after exposing to cadmium for 0 (as control), 6, 12, and 24 h, respectively. The results (Fig. 8) showed that all of genes expression was significantly disturbed under cadmium treatment except *BcaCPK6.B02*. With elongated treated time, among these genes, the expression of two genes (*BcaCPK2.B01a* and *BcaCPK2.B01a*) was decreasing and one gene increasing (*BcaCPK5.B02a*), while the expression of remaining genes exhibited dynamic change at different point under cadmium stress.

### Expression profiles of BcaCPKs under salt treatment using qRT-PCR

**Fig. 9:**
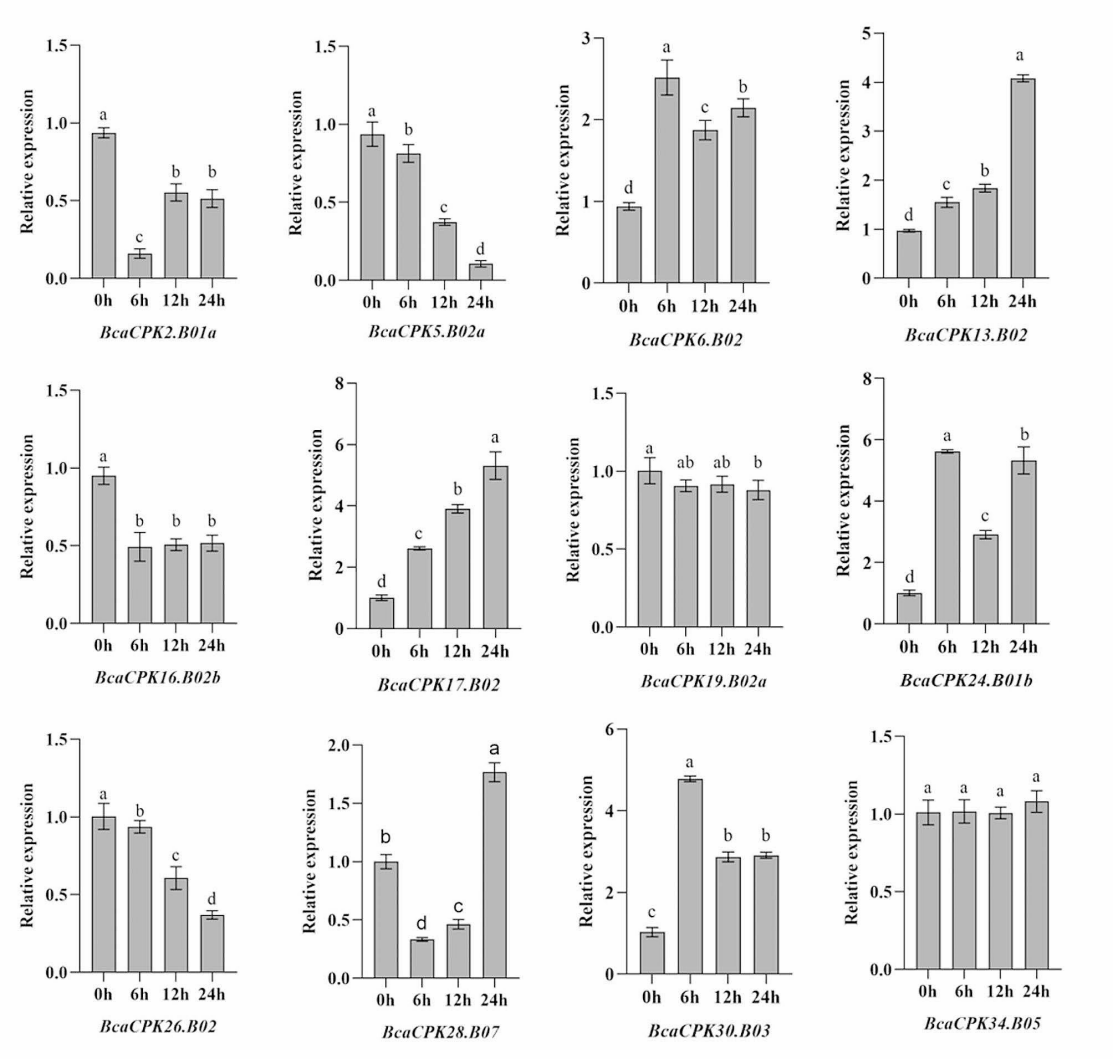
The relative expression of selected *BcaCPK*s under the treatment of 150mM/L NaCl. Different letters indicate a statistically significant difference at *P* < 0.05

To detect the expression profile of *BcaCPKs* in responding to salt stress, the relative expression of above genes at different point under salt stress was also measured using qRT-PCR (Fig. 9). The results showed that, compared to control, the expression levels of these selected *BcaCPKs* was significantly affected under salt treatment except for *BcaCPK19.B02* and *BcaCPK34.B05*. We noticed that *BcaCPK19.B02* and *BcaCPK34.B05* were significantly upregulated under salt treatment, suggesting that the two genes likely involved in responding to salt stress. Furthermore, we found that the expression of *BcaCPK24.B01b*, *BcaCPK28.B07*, and *BcaCPK30.B03* were significantly induced under both cadmium and salt treatments, while the expression levels of *BcaCPK2.B01a*, *BcaCPK16.B02b*, and *BcaCPK26.B02* were decreased under both stresses, indicating that the *BcaCPKs* likely played an important role in abiotic stress.

## Discussion

*B. carinata* belongs to the Brassicaceae *Brassica* crop. It is one of the three composite species of the “U” triangle. It is considered to be derived from the doubling of chromosomes under natural conditions after hybridization between *B. oleracea* and *B. nigra* [16]. The occurrence of polyploidization events often strengthens heterosis and forms new polyploids to adapt to more complex environmental conditions. In addition, the formation of polyploids has a profound impact on ecology and plant evolution. Polyploidization can enhance plant tolerances, such as drought tolerance, salt tolerance, high temperature resistance, pest resistance, and so on [24, 25]. *B. carinata* was first discovered and planted in countries such as Ethiopia and Sudan. It has a long history of cultivation, dating back to 4000 BC. It plays a very important role in agricultural production. Seeds can be used to make condiments, which have been planted and studied in many countries and regions [26]. *B. carinata* has many ideal agronomic traits, such as heat tolerance, drought tolerance, salt tolerance, lodging resistance and pest resistance [27–30]. These characteristics allow it to adapt to a wider range of environmental conditions than other *Brassica* plants. Recently, *B. carinata* has received increasing attention as an energy crop, and the cultivation of biofuel crops in Canada and the United States has increased significantly over the past decade [31]. More importantly, *B. carinata* can grow in extremely harsh environments, such as hot, arid or semi-arid areas, but these environments are not suitable for the survival of *B. napus* [28, 32]. Calcium-dependent protein kinases (CPKs) are a class of serine/threonine protein kinases that are widely present in plants. They have dual functions of Ca^2+^ sensor and responder, which can regulate plant growth and development and induce protective responses to environmental stress [4, 33]. It is encoded by a multi-gene family and was first discovered in *Pisum sativum* by Hetherington et al. [34]. It has been identified in a variety of plants so far, but rarely studied in *B. carinata*. In 2022, the genome sequencing data of *B. carinata* was published [20]. The analysis of the *CPK* gene family of *B. carinata* at the genomic level can provide a reference for promoting the development of molecular breeding of *B. carinata*.

In this study, based on the *(A) thaliana AtCPK* sequence and its typical protein domain Ser/Thr protein kinase region and EF-hand structure region, 123 *BcaCPK* genes were identified in the *(B) carinata* genome database by constructing HMM and BLAST. The study of *CPK* gene family from green algae to terrestrial plants showed that *CPK* gene family was more conservative in terrestrial plants, and CPK in monocots and dicots was conservatively divided into four subfamilies [35]. In this study, the phylogenetic tree of *B. carinata* and *A. thaliana* can be divided into four subfamilies, which is consistent with the results of other plants, indicating that there are still some commonalities among species [36, 37]. Among them, the number of Clade IV was the largest (45), and the number of Clade II was the least (16). CPK has obvious structural characteristics in plants. In this study, it was found that the length of the N-terminal variable region of 123 *BcaCPK* members was different. The catalytic domain protein kinase region has high homology and contains a typical Ser/Thr protein kinase catalytic conserved sequence; the regulatory region contains four EF-hand structures. Based on the previous research progress, it is speculated that the *CPK* gene may come from the fusion the protein kinase and CaM gene. However, gene structure analysis found that the number of introns and exons of *BcaCPK* members was quite different. This may be an important reason why *BcaCPK* gene family members play different roles in plants. The distribution of introns and exons in genes and the number of introns are typical evolutionary markers of plant gene families [38]. In eukaryotes, introns are spliced by exons, and the increase in the number of introns enriches the type of genes and the function of proteins [39]. Therefore, it is reasonable to speculate that the evolution of *BcaCPK* gene structure is driven by changes in the number of introns, and this change may be related to different environmental stresses.

In this study, there were 52 *CPK* genes in *B. oleracea* and *B. nigra*, respectively. 123 *BcaCPK* genes were unevenly distributed on 34 chromosomes of *B. carinata*, and the *CPK* gene family was significantly expanded during the formation of the species. The number of *CPK* genes in the B subgenome of allotetraploid *B. carinata* was the most variable (52 *BniCPK* genes in the B genome and 70 *BcaCPK* genes in the B subgenome), and the number of *CPK* genes in the C subgenome was almost unchanged (52 *BniCPK* genes in the C genome and 53 *BcaCPK* genes in the C subgenome), which may be affected by genetic variation during the evolution of *B. carinata*. Gene duplication event is another important evolutionary mechanism, which provides a potential possibility for the change of gene function, leading to the evolution of genes [40]. The divergence between the common ancestors of *Brassica* and *Arabidopsis* began about 20 million years ago. Subsequently, approximately 16 million years ago, the *Brassica* ancestors experienced a genome-wide replication event [41, 42]. In addition, dispersed duplication or tandem duplication can also lead to an increase in the number of genes [43, 44]. There are 120 repetitive events in the *B. carinata* genome, including 8 tandem duplications and 112 dispersed duplications, indicating that dispersed duplication events are the main reason for the expansion of the *BcaCPK* gene family. Among them, 99 pairs of *CPK* gene pairs with collinear relationships had Ka/Ks less than 0.5, indicating that the *CPK* gene of *B. carinata* was very conservative in evolution and conservatively mutated under the pressure of purification selection.

The *cis*-acting elements related to stress were found in the promoter region of most *BcaCPK* genes, such as ABRE, MBS and LTR. On the other hand, this result confirmed that the *BcaCPK* gene may be involved in the response of *B. carinata* to various abiotic stresses. Studies have shown that CPK proteins are involved in a variety of biological processes in plants (such as growth and development, resistance to biotic and abiotic stresses) and are widely distributed in plant tissues [45]. The expression of *BcaCPK24.B01b*, *BcaCPK28.B07*, and *BcaCPK30.B03* was significantly up-regulated under cadmium and salt stress, while the expression of other members was significantly up-regulated under at least one stress, suggesting that these genes may be involved in the regulation of cadmium and salt stress. The specific gene function needs to be verified by transformation experiments. These results suggest that *BcaCPK* genes are involved in the regulation of cadmium and salt stress signals through different molecular mechanisms. In this study, although the phylogenetic evolution, gene structure and expression analysis of the *CPK* gene family of *B. carinata* were carried out, it was preliminarily verified that the CPK gene had an expression level response to cadmium and salt treatment in *B. carinata*. However, the function of *BcaCPK* in cadmium and salt stress in *B. carinata* needs to be further studied. This study provides a reference for the next step of gene cloning and transformation, functional verification and molecular marker-assisted breeding marker development.

## Materials and methods

### Identification of CPK gene family members in B. carinata

The CPK protein sequence of *Arabidopsis thaliana* was obtained from the TAIR (*Arabidopsis thaliana*, 2000) database available at https://www.arabidopsis.org. The hidden Markov model (HMM) of the core protein kinase domain (PF00069) and EF-hand domain (PF13499) of CPK family genes were also downloaded. To identify the *CPK* gene family members, the following steps were followed: (1) The *Arabidopsis* CPK protein sequence was used to perform a local blast against the *B. carinata* genome data (http://brassicadB.org/brad/) [46], with an E value threshold of 1e-5; (2) The sequences containing both the EF-hand motif and Pkinase domain were screened using HMMER 3.0 software [47], with domE value of 1e-5. Based on the above screening results, the repeat sequence was removed, and the domain was verified using the SMART database (http://smart.embl-heidelberg.de) [48]. The remaining sequences were identified as members of the *CPK* gene family of *B. carinata*. To analyze and predict various parameters of the CPK protein sequence, the online tool ExPASy Proteomics Server was utilized. The EF-hand structure was identified using the online tool PROSITE (https://prosite.expasy.org/prosite.html), while the CSS-Plam program was employed for palmitoylation site prediction. Additionally, online tool Myristoylation (https://web.expasy.org/myristoylator/) was used to predict the myristoylation site. The subcellular localization of the CPK protein in *B. carinata* was predicted using the Cell-PLoc 2.0 online software [49].

### Phylogenetic analysis of CPK sequences in B. carinata and A. thaliana

The MUSCLE program was utilized to compare 123 full-length protein sequences of CPK in *B. carinata* with 34 CPK protein sequences in *A. thaliana* under default parameters [50]. Then, the phylogenetic tree of CPK protein was constructed using MGEA11 v11.0.13 [51] software, employing the ML method (1000 bootstrap replicates) combined with the Tamura-Nei nucleotide substitution model. To ensure the stability of the phylogenetic tree, the bootstrap method was employed with 1000 repetitions. Finally, the phylogenetic tree was annotated using the web service of (iTOL) v5 (httpt://itol.embl.de/) [52].

### Analysis of BcaCPKs structure and conserved motifs

To predict the conserved motifs of its protein sequence, the MEME online tool (https://meme-suite.org/meme/index.html) [53] was employed. Additionally, the conserved domain information of the BcaCPK family was analyzed using NCBI-CDD (https://www.ncbi.nlm.nih.gov/cdd/). To understand the *BcaCPK* gene structure, we employed GSDS 2.0 software (Gene Structure Display Server 2.0, http://gsds.cbi.pku.edu.cn//index.php) [54] to detect the exon/intron composition information based on the default parameters.

### Chromosomal localization analysis of BcaCPKs

The physical distribution information of *CPK* genes in *B. carinata*, *B. nigra* and *B. oleracea* was obtained from the genome annotation files of *Brassica* BC genome. Chromosome localization of *CPKs* gene family members in *Brassica* BC genome was carried out by using the gene localization visualization function of TBtools software [55].

### Gene duplication, collinearity, and evolutionary analysis of BcaCPKs

Gene duplication that has been a common phenomenon in plant species plays a significant role in the expansion of gene families. In this study, we utilized Dup Gen _finder to analyze the repetitive events of the *CPK* gene. Subsequently, the collinear gene pairs of the *CPK* gene family in *B. carinata* were identified using JCVI. To align the *BcaCPKs*, PRANK was employed, and the NG model in KaKs_Calculator 2.0 was utilized to estimate values of Ka, Ks, and Ka/Ks which provides insights into the selection pressure on repetitive gene pairs.

### Analysis of cis-acting elements (CREs) in promoter region of BcaCPKs

To identify the CREs of *BcaCPKs*, TBtools was utilized to extract a 2000 bp upstream of the promoter of these *BcaCPKs* coding sequence from the genome of *B. carinata*. The online database PlantCARE (http://bioinformatics.psb.ugent.be/webtools/plantcare/html/) was employed to predict the *cis*-acting elements of interest [56].

### Gene expression profiles of BcaCPKs under salt and Cd stress

The data of gene family tissue expression analysis were derived from our previous experimental data and gene transcriptome analysis was performed with the *B. carinata* genome as a reference. The *B. carinata* pure line with purple leaf was used in this study. The seeds of this line were cultured in a petri dish with filter paper for one week. Seedlings with vigorous growth and uniform state were selected and cultured in Hoagland nutrient solution for two weeks. They were then subjected to cadmium and salt stress, respectively. The control group was cultured in normal Hoagland’s nutrient solution, while the treatment groups T1 and T2 were treated with Hoagland’s nutrient solution containing 150 mM/L NaCl and 150 mM/L CdCl_2_, respectively. After under the treatment of 0, 6, 12, and 24 h, samples were frozen in liquid nitrogen and RNA was extracted from the aboveground part using an RNA extraction kit (Aidlab Biotech, Beijing, China). The expression of *BcaCPKs* under salt and cadmium stress was determined using qRT-PCR. The leaves treated with 0 h served as the control (Control), and the relative expression of the gene in each period was calculated using the 2^−△△CT^ method [57]. Primers of these selected genes were designed according to Primer Premier 5, and their specificity was verified within the genome using TBtools software [55]. (Table S6), the actin is designed according to the articles published [32].

### Statistical analysis

Statistical analysis of data in this study was determined by conducting a one-way analysis of variance (*P* < 0.05) using SPSS 13.0 (SPSS Inc., Chicago, IL, USA). All data are presented as the mean ± standard error based on at least three independent biological replicates.

## Conclusions

In this study, the *CPK* gene family was comprehensively identified and analyzed in the *B. carinata* genome, and its evolutionary relationship, gene structure, replication events and *cis*-acting elements were systematically analyzed. A total of 123 *CPK* genes were identified in the *B. carinata* genome, all of which have serine/threonine protein kinase and EF-hand domains. By constructing a phylogenetic tree, four subfamilies were obtained. The members within the subfamily are highly conserved, while the sequence and structural characteristics of the members of each subfamily are different, and the members of each subfamily have a similar number of gene structures. The distribution of 123 *BcaCPK* genes on 34 chromosomes of *B. carinata* was uneven. Dispersed duplication is the way of *BcaCPK* gene family amplification, which is mainly affected by purification selection in evolutionary selection. The *cis*-acting elements of the promoter of each gene member were predicted that most of them contained multiple hormone response elements and stress response elements. The results of qRT-PCR analysis showed that the expression of *BcaCPK* gene was significantly different under different abiotic stresses, indicating that *BcaCPK* gene played an important role in the response of *B. carinata* to different abiotic stresses. The results provide relevant information for the subsequent exploration of the response mechanism of the CPK family in *B. carinata* to the external abiotic stress environment.

### Electronic supplementary material

Below is the link to the electronic supplementary material.

Supplementary Material 1

## Data Availability

All data generated or analyzed during this study were included in this published article and the additional files.
